# The expression of P-glycoprotein does influence the distribution of novel fluorescent compounds in solid tumour models

**DOI:** 10.1038/sj.bjc.6601300

**Published:** 2003-10-14

**Authors:** C Martin, J Walker, A Rothnie, R Callaghan

**Affiliations:** 1 Nuffield Department of Clinical Laboratory Sciences, John Radcliffe Hospital, University of Oxford, Oxford OX3 9OU, UK

**Keywords:** drug distribution, P-glycoprotein, tumour models, confocal fluorescence microscopy, drug resistance

## Abstract

Solid tumours display a complex drug resistance phenotype that involves inherent and acquired mechanisms. Multicellular resistance is an inherent feature of solid tumours and is known to present significant barriers to drug permeation in tumours. Given this barrier, do acquired resistance mechanisms such as P-glycoprotein (P-gp) contribute significantly to resistance? To address this question, the multicellular tumour spheroid (MCTS) model was used to examine the influence of P-gp on drug distribution in solid tissue. Tumour spheroids (TS) were generated from either drug-sensitive MCF7^WT^ cells or a drug-resistant, P-gp-expressing derivative MCF7^Adr^. Confocal microscopy was used to measure time courses and distribution patterns of three fluorescent compounds; calcein-AM, rhodamine123 and BODIPY-taxol. These compounds were chosen because they are all substrates for P-gp-mediated transport, exhibit high fluorescence and are chemically dissimilar. For example, BODIPY-taxol and rhodamine 123 showed high accumulation and distributed extensively throughout the TS^WT^, whereas calcein-AM accumulation was restricted to the outermost layers. The presence of P-gp in TS^Adr^ resulted in negligible accumulation, regardless of the compound. Moreover, the inhibition of P-gp by nicardipine restored intracellular accumulation and distribution patterns to levels observed in TS^WT^. The results demonstrate the effectiveness of P-gp in modulating drug distribution in solid tumour models. However, the penetration of agents throughout the tissue is strongly determined by the physico-chemical properties of the individual compounds.

Conventional chemotherapy continues to remain at the front line of treatment strategies against cancer and the last two to three decades have witnessed an ever-increasing armoury of clinically useful compounds (Skeel, 1999; Baguley, 2002). Unfortunately, a substantial proportion of chemotherapy treatments fail due to the myriad of drug resistance pathways generated by most forms of cancer (Chaney and Sancar, 1996; Wang *et al*, 1999; Desoize and Jardillier, 2000; Gottesman *et al*, 2002). Resistance pathways may be broadly characterised as affecting the pharmacodynamics (response) or pharmacokinetics (lifetimes and exposure) of anticancer agents. Pharmacodynamic resistance pathways include factors such as altered drug target sensitivity, increased DNA repair pathways and a reduced ability to produce an apoptotic response. Pharmacokinetic pathways produce alterations in the stability, metabolism, excretion and distribution of drugs at the tumour site. Thus, the situation *in vivo* is clearly more complex than can be explained by any single factor such as the much-touted multidrug resistance pump P-glycoprotein (P-gp) (Gottesman *et al*, 2002).

P-glycoprotein is a member of the ABC superfamily of transporters (Holland and Blight, 1999) and is able to confer resistance by actively extruding an extraordinarily diverse range of chemotherapeutic agents and a significant amount of research effort has been directed towards understanding and circumventing the actions of P-gp *in vivo*. But has the contribution of P-gp to drug resistance warranted this attention? In the case of haematological malignancies (e.g. AML, ALL, multiple myeloma and non-Hodgkin's lymphoma), the protein is frequently expressed both prior to, and following, exposure to chemotherapeutic regimes (for a review see Sonneveld, 1996; Chauncey, 2001). Expression of P-gp in such cancers is associated with an increased likelihood of relapse, fewer remissions and a poor survival time (Willman, 1997; van den Heuvel-Eibrink *et al*, 2000; Dhooge *et al*, 2002). Strategies to circumvent the drug resistance phenotype in haematological tumours by inhibition of P-gp have met with some success and compounds such as PSC833, LY79553 and XR9576 have entered clinical trials (Fields *et al*, 1998; Stewart *et al*, 2000). However, the situation is less clear in solid tumours. Certainly, the protein is endogenously expressed in a variety of tumours (for a review see Goldstein, 1996). Furthermore, the expression is frequently induced or upregulated following chemotherapy particularly in breast and the gastrointestinal tract (Schneider *et al*, 1989; Ro *et al*, 1990; Chan *et al*, 1991,^1997^; Pirker *et al*, 1993; Tokunaga *et al*, 2001; Coley *et al*, 2002).

However, inhibition of P-gp in many solid tumours has not always been associated with improved clinical outcome. The results have been interpreted in terms of (i) inefficient P-gp inhibition, (ii) the involvement of other resistance pathways and/or (iii) a lack of clinical relevance for P-gp. The latter argument stems partly from the knowledge that solid tumours present a significant inherent barrier to drug pharmacokinetics, even without P-gp expression. The complex 3-D arrangement of avascular regions (or nodes) situated between capillaries are the primary target regions for chemotherapeutic agents (Desoize and Jardillier, 2000; Mueller-Klieser, 2000) and display a hostile environment (Folkman, 1971; Harris, 2002) that impairs chemotherapy by altering drug response either inherently or by increasing the expression of specific proteins (see Harris, 2002). Furthermore, drug pharmacokinetics within the avascular regions will be attenuated by the high local interstitial pressure, extensive cell–cell contacts and the extracellular matrix (Desoize and Jardillier, 2000; Mueller-Klieser, 2000). P-glycoprotein expression would be expected to affect intracellular accumulation; however, the influence on overall tissue distribution is unclear. The influence of inherent multicellular barriers has persuaded many clinicians, and some scientists, to relegate the role of P-gp to almost bystander status. Can we really have that much confidence in disregarding the influence of this archetypal ‘multidrug’ transporter on the pharmacokinetics of anticancer agents in solid tumours?

To address this, the versatile multicellular spheroid (MCTS) model (Kunz-Schughart *et al*, 1998; Santini and Rainaldi, 1999) was chosen to investigate the influence that P-gp exerts on drug distribution in solid tumours. In order to examine drug distribution patterns within tumour spheroids (TS), a confocal fluorescence microscopy technique was employed, a particular advantage being the ability to measure drug distribution in intact tissue. The TS were formed from either the drug sensitive MCF7^WT^ breast cancer cell lines or a P-gp-expressing drug-resistant derivative. Distribution profiles were characterised for three fluorescent compounds that are transported substrates of P-gp. The results provide insight into the drug-specific effects of P-gp on pharmacokinetic properties within a solid tissue mass.

## MATERIALS AND METHODS

### Materials

Dulbecco's minimum essential medium (DMEM) with GlutaMax I, penicillin, streptomycin and foetal calf serum were purchased from Invitrogen (Paisley, UK). BODIPY® FL paclitaxel (BODIPY-taxol) and calcein-acetoxymethyl ester (calcein-AM) were obtained from Molecular Probes (Leiden, The Netherlands). Nicardipine and rhodamine123 were purchased from Sigma-Aldrich (Poole, UK). [^3^H]azidopine (51 Ci mmol)^−1^ was from Amersham Biosciences, Amersham, UK. *Escherichia coli* (*E. coli*) phospholipids and cholesterol were purchased from Avanti Polar Lipids (AL, USA) and octyl-*β*-D-glucoside was from Merck Biosciences (Nottingham, UK).

### Cell culture and TS growth

Drug-sensitive (MCF7^WT^) human breast cancer cells were obtained from the NCI-Frederick cancer DCTD Tumour cell repository. The drug-resistant (MCF7^Adr^) cells were obtained from Professor Cowan and were generated by selection in adriamycin, as described (Batist *et al*, 1986), from MCF7^WT^ cells (Soule *et al*, 1973). Both cell lines were grown as monolayer cultures in DMEM supplemented with 10% (v v^−1^) foetal calf serum and penicillin/streptomycin (100 IU ml^−1^ and 100 mg ml^−1^, respectively). The resistant MCF7^Adr^ cell line was cultured in the presence of 3 *μ*M doxorubicin for a single passage every 10 passages to maintain selection pressure.

Tumour spheroids of MCF7 cell lines were grown using the liquid overlay technique (Kunz-Schughart and Meuller-Klieser, 2000) in 96-well tissue culture plates. The 96-well plates were given a 100 *μ*l base-coat of 0.75% (w v^−1^) agar that had been prepared in DMEM. Freshly trypsinised MCF7 cells were overlaid on solid agar base-coats at a density of 4 × 10^3^ cells in a volume of 200 *μ*l DMEM. The MCF7^WT^ cell lines were kept still for 24 h (37°C, 5% CO_2_) after which the plates were shaken at 300 r.p.m. in a tissue culture incubator. Tumour spheroids generated from MCF7^WT^ cells will be referred to as TS^WT^ and similarly, those from MCF7^Adr^ cells as TS^Adr^ throughout the manuscript. MCF7^Adr^ cell overlays were kept stationary for 48 h prior to shaking due to a greater fragility at early stages of growth. TS^WT^ were fully formed within 48 h and could be routinely cultured under these conditions for up to 10 days, with replacement of the medium at 3-day intervals. In contrast, TS^Adr^ required 72 h to form fully.

### Expression and purification of P-gp

Human P-gp was expressed in insect cells using the baculovirus expression system and full details of the purification procedure for P-gp have been reported previously (Taylor *et al*, 2001). The purified protein was reconstituted into liposomes comprising a 4 : 1 (w w^−1^) mixture of crude *E. coli* phospholipids and cholesterol at a protein : lipid ratio of 50 (w w^−1^) to allow functional assessment. The protein concentrations were in the range 15–20 *μ*g ml^−1^ and proteoliposomes could be stored in buffer comprising 150 mM NaCl, 20 mM Tris pH 7.4, 1.5 mM MgCl_2_, 20% (v v^−1^) glycerol buffer for up to 6 months at −80°C.

### Photoaffinity labelling of P-gp with [^3^H]-azidopine

Reconstituted P-gp was labelled with the photoactivatable inhibitor [^3^H]azidopine according to previously published procedures (Taylor *et al*, 2001). Briefly, the proteoliposomes (250 ng) were incubated in the dark with 0.5 *μ*M [^3^H]azidopine in the presence or absence of bodipy-taxol (5 *μ*M), rhodamine 123 (10 *μ*M) or calcein-AM (10 *μ*M) for 1 h at 20°C. The total incubation volumes were 40 *μ*l. The unreacted [^3^H]azidopine was separated by 8% SDS–PAGE and the band intensity was quantified by densitometry (NIH 2.0 Image Software) of autoradiograms.

### ATPase activity of P-gp

The ATPase activity of purified P-gp was determined using modifications (Taylor *et al*, 2001) of the previously described colorimetric assay (Chifflet *et al*, 1988) to measure the liberation of free inorganic phosphate. The proteoliposomes (250 ng) were incubated with 2 mM ATP in the absence or presence of 10 *μ*M nicardipine to determine the maximal rates of basal and drug-stimulated activity, respectively. The total sample volume was 50 *μ*l and incubations were for 20 min at 37°C (Callaghan *et al*, 1997). The effects of bodipy-taxol (5 *μ*M), rhodamine 123 (10 *μ*M) or calcein-AM (10 *μ*M) on ATPase activity were determined during measurement of both basal and nicardipine-stimulated activity. All drug additions were from concentrated DMSO stocks and the solvent concentration did not exceed 1% (v v^−1^).

### Confocal fluorescence microscopy of TS and monolayers

The distribution of calcein-AM, BODIPY-taxol and rhodamine123 in TS with diameters in the range 300–400 *μ*M was studied using a Zeiss LSM510 confocal laser scanning microscope (Carl Zeiss, Welwyn Garden City, UK). All three fluorophores were excited using an argon laser (excitation wavelength=488 nm) and detected using an emission filter set at 505–530 nm. Tumour spheroids were exposed to BODIPY-taxol (2.5 *μ*M), calcein-AM (5 *μ*M) or rhodamine 123 (5 *μ*M) at 37°C for periods indicated in the text and figure legends. Following incubation, the TS were washed in buffer A (150 mM NaCl, 20 mM Tris pH 7.4) and placed in cavity microscope slides (RA Lamb, Sussex, UK). Where P-gp inhibition was required, nicardipine (10 *μ*M) was added for a preincubation period of 1 h prior to the addition of fluorescent compounds. To examine the 3-D distribution of fluorophores within TS, a scan in the *z*-direction was performed at 4 *μ*M steps over a maximum depth of 80 *μ*M from the periphery of the tissue to provide 20 discrete images. The intensity was measured along two perpendicular lines that bisected the image along the *x*- and *y*-axis. The signal was averaged for data obtained from the two lines to provide an averaged measure of fluorescence intensity along the ‘*xy*-plane’.

Drug accumulation measurements in monolayers required that cells were grown on coverslips. The coverslips were washed in PBS and placed in buffer A containing fluorescent compounds at concentrations stated above. Inhibition of P-gp function was achieved, where required, with a preincubation of cells in buffer A containing nicardipine (10 *μ*M) for a preincubation period of 1 h. The coverslips were washed in PBS and covered with a thin film of buffer A containing 20% (v v^−1^) glycerol. The coverslips were inverted and placed on microscope slides and the edges sealed with clear nail varnish. Drug accumulation was measured in the monolayers using fluorescence confocal microscopy as described above. The optical sections obtained along the *z*-axis were achieved using 1 *μ*M increments.

## RESULTS

### Verification of BODIPY-taxol as a substrate for P-gp

**Figure 1:**
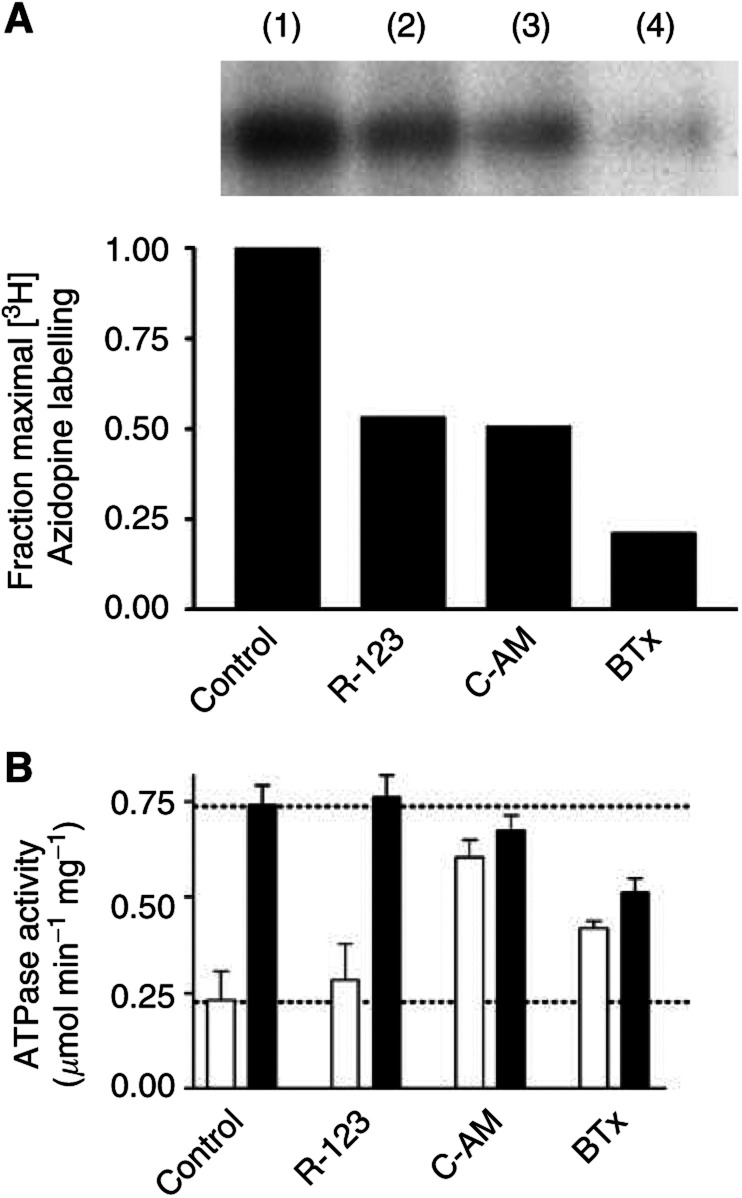
Azidopine displacement by model fluorescent substrates of P-gp. (**A**) Purified, reconstituted human P-gp (250 ng) was incubated with [^3^H]azidopine (0.5 *μ*M) for 60 min in the absence (lane 1) or presence of 10 *μ*M rhodamine 123 (lane 2), 10 *μ*M calcein-AM (lane 3) or 5 *μ*M BODIPY-taxol (lane 4). The samples were then placed on ice and irradiated (315 nm, 100 W at 5 cm) for 5 min. Protein was separated from unbound [^3^H]azidopine by SDS–PAGE and subjected to autoradiographic analysis. (**B**) The ATPase activity of purified, reconstituted human P-gp (250 ng) was determined by measurement of liberated inorganic phosphate. Basal and drug-stimulated (10 *μ*M nicardipine) ATPase activities were determined in the absence or presence of rhodamine 123 (10 *μ*M), calcein-AM (10 *μ*M) or BODIPY-taxol (5 *μ*M). Basal and nicardipine-stimulated activities are represented by clear and filled bars, respectively. Error bars denote the s.e.m. and the dotted lines represent the level of basal and stimulated activity in the absence of fluorescent allocrite.

Several previous investigations have characterised the distribution of the inherently fluorescent P-gp substrate doxorubicin in solid tumours. However, the fluorescence of doxorubicin is relatively weak and several alternative compounds with which to assess drug distribution and the influence of P-gp on drug pharmacokinetics are available. For example, calcein-AM and rhodamine 123 were included, since they are well-characterised allocrites for P-gp and display high fluorescence intensity (Homolya *et al*, 1993; Shapiro and Ling, 1998). BODIPY-taxol, a novel fluorescent derivative of the anticancer drug paclitaxel has been suggested, but not proven, to interact with P-gp (Fellner *et al*, 2002). Taken together, the data presented in Figure 1

obtained using purified and reconstituted P-gp demonstrate that BODIPY-taxol is indeed capable of direct interaction with this transporter. [^3^H]Azidopine was used to photoaffinity label P-gp (Figure 1A), and a 0.5 *μ*M concentration was employed to ensure reasonable saturation of the protein, given that this drug was previously shown to display a *K*_d_ of 450 nM (Taylor *et al*, 2001). All the three fluorescent compounds, at the indicated concentrations, were able to reduce the photoaffinity labelling of P-gp by azidopine. Calcein-AM and rhodamine 123 at concentrations of 10 *μ*M produced a reduction in [^3^H]azidopine labelling of approximately 50% (Figure 1A). BODIPY-taxol produced the greatest inhibition of P-gp labelling with only 20% of the signal remaining in the presence of this paclitaxel derivative.

To determine the consequence(s) of interaction of these fluorescent compounds with P-gp, the effects on ATP hydrolysis were measured (Figure 1B). The basal ATPase activity of P-gp (0.23±0.07 *μ*mol Pi min^−1^ mg^−1^) was stimulated 3.2-fold by nicardipine to a maximal rate of 0.74±0.05 *μ*mol Pi min^−1^ mg^−1^. Rhodamine 123 (10 *μ*M) did not affect either the basal or nicardipine-stimulated activity. In contrast, calcein-AM caused a 2.6-fold increase in basal ATPase activity to a value of 0.60±0.05 *μ*mol Pi min^−1^ mg^−1^. There was a marginal reduction in the level of nicardipine-stimulated activity; however, this did not reach a statistically significant value. BODIPY-taxol caused a 1.8-fold stimulation of the basal activity to 0.42±0.02 *μ*mol Pi min^−1^ mg^−1^ and this degree of stimulation is similar to that produced by paclitaxel (Gabriel *et al*, 2003). The coincubation of P-gp with BODIPY-taxol (5 *μ*M) and nicardipine (10 *μ*M) resulted in a reduced degree of stimulation compared to that solely produced by nicardipine. Thus, under these conditions BODIPY-taxol acts as a partial agonist on ATP hydrolysis and modifies the actions of nicardipine. Taken together, the photoaffinity labelling and ATPase activity data indicate that BODIPY-taxol, like calcein-AM and rhodamine 123, is capable of direct interaction with P-gp.

### Cellular accumulation of BODIPY-taxol and calcein-AM

**Figure 2:**
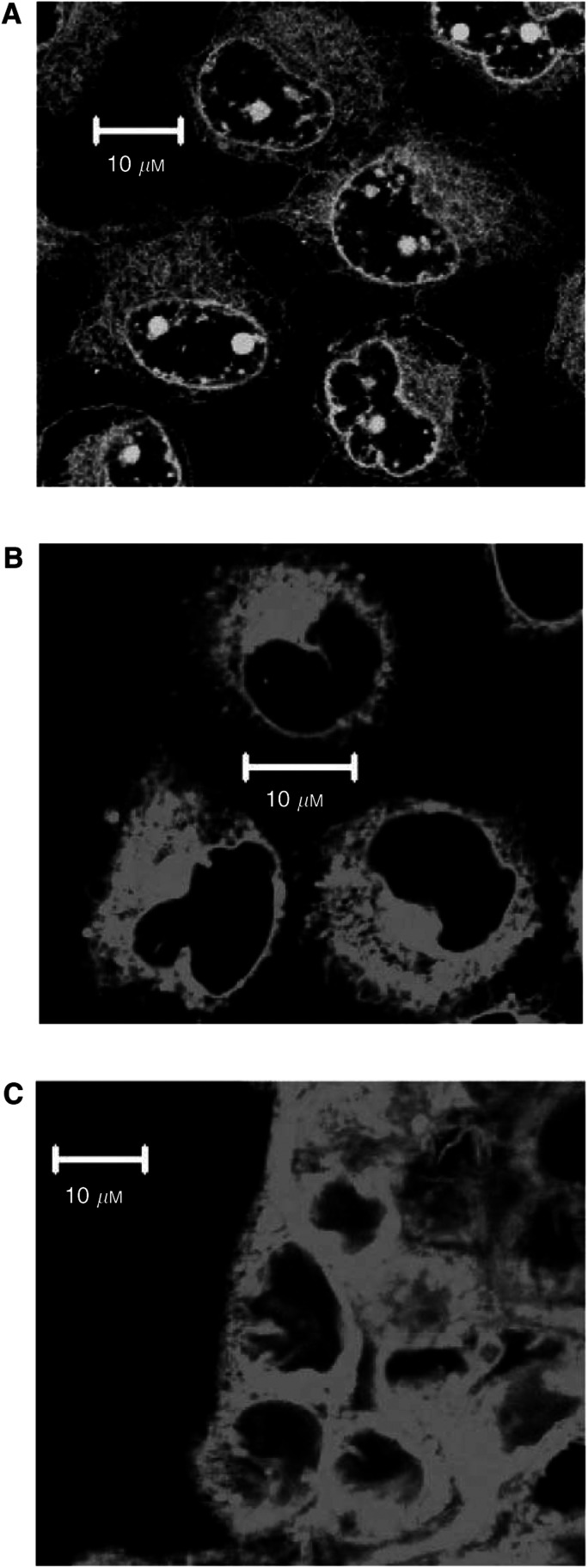
Accumulation of P-gp substrates in MCF7^WT^ cells and TS^WT^. The accumulation of 5 *μ*M calcein-AM was determined in (**A**) monolayer cultures of MCF7^WT^ cells. The cellular levels of 2.5 *μ*M BODIPY-taxol were also measured in (**B**) monolayer or (**C**) TS systems. The fluorescent compounds were incubated with cells for 60 min at 37°C in a humidified atmosphere (5% CO_2_). The images were obtained by confocal fluorescence microscopy and for TS they were taken from cells close to the tissue periphery.

The investigations reported above do not demonstrate whether BODIPY-taxol is able to cross cellular membranes, thereby entering a ‘compartment’ susceptible to extrusion by P-gp. Should BODIPY-taxol readily cross the cellular membrane, it would fulfil all the necessary criteria allowing its use as a probe to examine the effects of P-gp on drug distribution in the TS model. The ability to cross the plasma membrane was initially examined in monolayer cultures of MCF7^WT^ cells for both BODIPY-taxol and calcein-AM. Calcein-AM was examined since it is known to possess high lipophilicity and is only converted to the fluorescent, membrane-impermeant derivative calcein by cytoplasmic esterases (Homolya *et al*, 1993). The intracellular localisation of calcein-AM was measured by confocal fluorescence microscopy and the results are shown in Figure 2

(panel A). The fluorescence observed is due to the calcein moiety, generated by nonspecific esterases, and is extensively distributed in the cytoplasm. However, an internal membrane is well defined and presumably corresponds to the nuclear envelope. In addition, there are several intensely fluorescent pockets that indicate intranuclear localisation. As shown in panel B (Figure 2), BODIPY-taxol is also able to cross the plasma membrane of MCF7^WT^ cells and accumulate within the cytosol to significant levels. An intracellular membrane was again well defined, however, in contrast to calcein-AM, there was no evidence of BODIPY-taxol accumulation within the nuclear compartment of these cells. There was negligible accumulation of either compound in the P-gp-expressing MCF^Adr^ cells using similar concentrations. However, following a 1 h preincubation in the presence of 10 *μ*M nicardipine, both drugs accumulated within MCF^Adr^ cells, producing high fluorescence intensity and displaying a similar distribution pattern to that observed in the parental cells (data not shown). The observations with BODIPY-taxol in both purified proteoliposomes and native cellular systems indicate that this compound is a useful tool to examine the effects of P-gp on drug distribution in solid tumour models.

### Time course of drug accumulation in MCF7^WT^ TS

**Figure 3:**
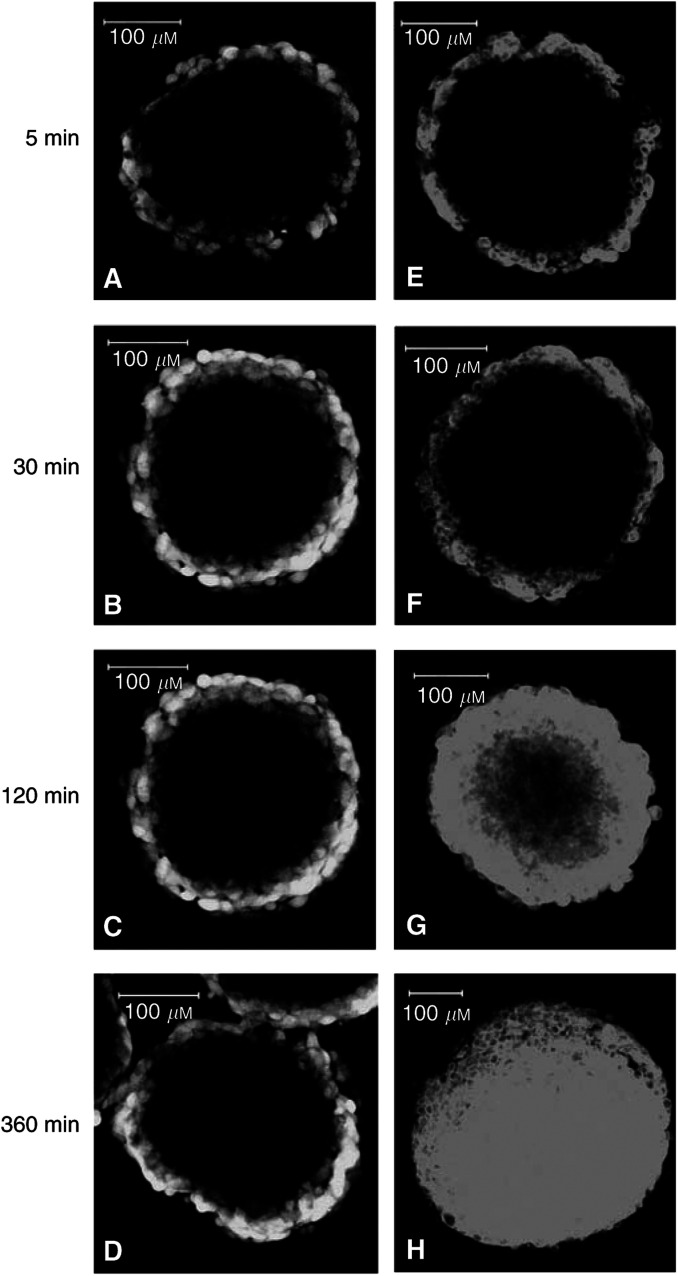
Time course for distribution of calcein-AM and BODIPY-taxol in TS. MCTS^WT^ (*d*=300–400 *μ*M) were incubated with 5 *μ*M calcein-AM (panels **A**–**D**) or 2.5 *μ*M BODIPY-taxol (panels **E**–**H**) for the periods indicated. The distribution of each compound was measured by confocal fluorescence microscopy and the images shown were taken at a depth of 60 *μ*M from the periphery along the *z*-axis. The white bars correspond to 100 *μ*M.

To characterise the intratissue distribution and intracellular drug accumulation in solid tissue not expressing P-gp, TS^WT^ (300–400 *μ*M) were exposed to BODIPY-taxol (2.5 *μ*M) and calcein-AM (5 *μ*M) over various time courses (Figure 3

). The distribution of drug was followed at a single depth (60 *μ*M) along the *z*-axis from the periphery of the tissue to enable determination of the comparative degree to which each compound was able to permeate the tissue. Moreover, this allowed assessment of whether drug could accumulate in quiescent cells in addition to the proliferating outer layers. Calcein-AM rapidly accumulated in the TS periphery as evidenced by the intense fluorescent signal observed following 5–30-min incubation (Figure 3, panels A, B). A striking observation was the lack of distribution in fluorescent signal to the central areas even following incubation of TS^WT^ in the presence of calcein-AM for up to 6 h (Figure 3, panels C, D). BODIPY-taxol accumulation in the peripheral layers of TS was also rapid, as evidenced by the similar degree of fluorescence intensity at 5 and 30-min incubation (Figure 3E–F). However, this paclitaxel derivative displayed a significantly different overall distribution pattern within TS^WT^ compared to that observed for calcein-AM. Longer durations of TS^WT^ exposure to BODIPY-taxol resulted in fluorescence distribution throughout the tissue rather than simply localised to the periphery (Figure 3G–H).

The fluorescent signal produced by BODIPY-taxol was examined at higher magnification to determine whether the distribution corresponded to intracellular or interstitial sites in TS^WT^. To ensure that significant intratissue distribution had occurred, the images were taken following 2-h incubation and at a depth of 60 *μ*M below the tissue periphery (see Figure 2C). The distribution pattern of fluorescence observed at this higher magnification demonstrates that BODIPY-taxol (Figure 2C) was accumulated intracellularly within a predominantly cytoplasmic localisation. Overall, the pattern of intracellular distribution of this paclitaxel derivative in TS^WT^ was similar to that observed in monolayer cultures. Subsequent investigations were aimed at determining whether the accumulation of drugs in cells within TS could be modulated due to the expression of P-gp.

### Drug accumulation and distribution in TS

**Figure 4:**
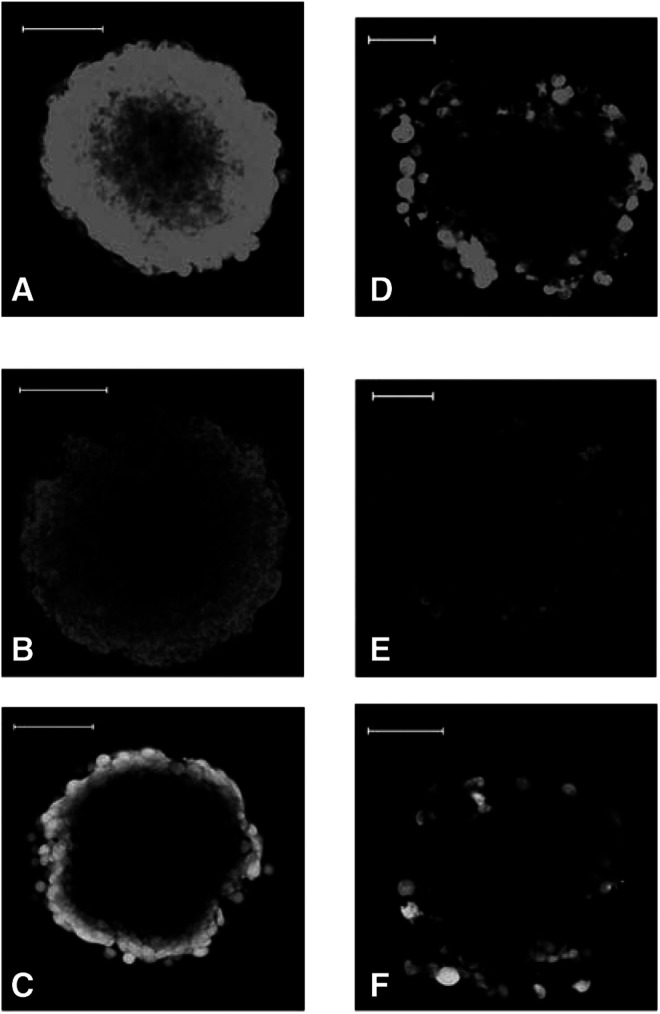
Permeation of drug-sensitive and -resistant TS by fluorescent allocrites of P-gp. TS^WT^ (panels **A**–**C**) and TS^Adr^ (panels **D**–**F**) were incubated in the presence of 2.5 *μ*M BODIPY-taxol (**A**, **D**), 5 *μ*M rhodamine-123 (**B**, **E**) or 5 *μ*M calcein-AM (**C**, **F**) for 60 min. Optical sectioning of TS (*d*=300–400 *μ*M) was achieved by confocal fluorescence microscopy and the images shown were taken at a depth of 60 *μ*M from the tissue periphery. The white bars correspond to 100 *μ*M.

The relative distribution of the fluorescent compounds within TS^WT^ or TS^Adr^ (*d*=300–400 *μ*M) is shown in Figure 4

with the optical sections obtained at a depth of 60 *μ*M from the tissue periphery. The results demonstrate that TS^Adr^ displayed a marked reduction in the overall accumulation of each of the compounds examined (Figure 4D–F) compared to that observed in the TS^WT^ (Figure 4A–C). Incubations of TS^Adr^ for periods up to 6 h did not enhance the intracellular accumulation or affect the overall distribution pattern (data not shown). The results provide direct evidence that the expression of P-gp in a solid tissue environment may impair the pharmacokinetics of its transported substrates by reduction in the intracellular accumulation. The obvious next question to tackle is whether this action of P-gp may be overcome pharmacologically.

**Figure 5:**
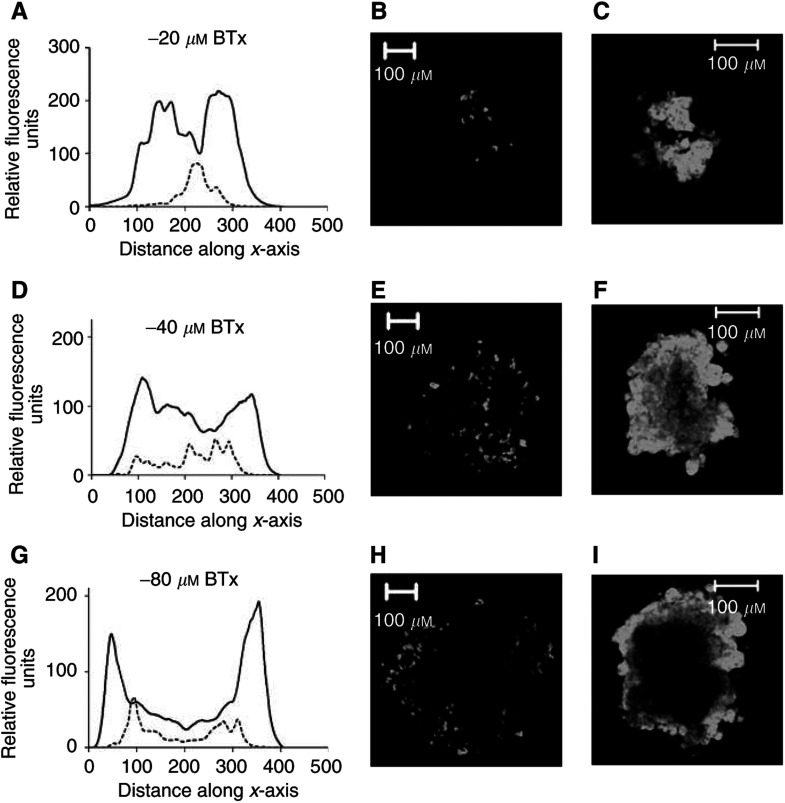
Cellular distribution of BODIPY-taxol in TS^Adr^. TS^Adr^ (*d*=300–400 *μ*M) were incubated with 2.5 *μ*M BODIPY-taxol for 60 min at 37°C in the presence or absence of 10 *μ*M nicardipine. Images were produced by optical sectioning to a depth of 80 *μ*M at 4 *μ*M increments along the *z*-axis of the tissue. The fluorescence intensity was measured for each of the images along the *xy*-axis. (**A**, **D** and **G**) intensity measured at depths of 20, 40 or 80 *μ*M from the periphery along the *z*-axis. (**B**, **E** and **H**) images taken in TS^Adr^ at depths of 20, 40 or 80 *μ*M along the *z*-axis (intensities are shown in the graphs as a dotted line). (**C**, **F** and **I**) images obtained from TS^Adr^ that had been incubated with 10 *μ*M nicardipine prior to addition of BODIPY-taxol (intensities shown in the graphs as solid lines).

**Figure 6:**
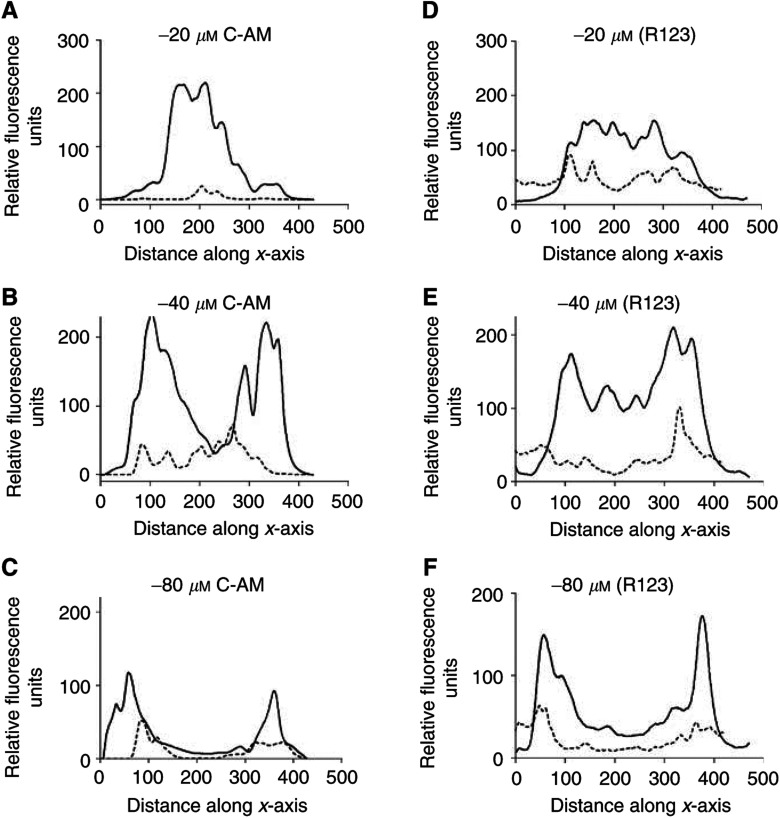
Quantitation of fluorophore distribution within TS^Adr^. TS^Adr^ (*d*=300–400 *μ*M) were incubated with 5 *μ*M calcein-AM or rhodamine 123 for 60 min at 37°C in the presence or absence of 10 *μ*M nicardipine. Images were produced by optical sectioning to a depth of 80 *μ*M at 4 *μ*M increments along the *z*-axis of the tissue. The fluorescence intensity was measured for each of the images along a central axis (*x*-axis). The data shown were taken from images at 20 *μ*M (**A**, **D**), 40 *μ*M (**B**, **E**) or 80 *μ*M (**C**, **F**). The dotted lines represent intensity of fluorescence produced by calcein-AM (**A**–**C**) or rhodamine 123 (**D**–**F**) in TS^Adr^, while the solid lines were obtained following incubation of these agents in the presence of 10 *μ*M nicardipine.

The distribution of each fluorescent compound was measured in TS^Adr^ to a depth of 80 *μ*M from the tissue periphery in increments of 4 *μ*M. The degree of BODIPY-taxol accumulation at three depths in the tissue (20, 40 and 80 *μ*M along the *z*-axis) is shown for TS^Adr^ in the presence (Figure 5C, F and I

) or absence of the P-gp inhibitor nicardipine (Figure 5B, E and H). The fluoresence was quantified at each depth and the intensity along the *xy*-plane of each image is shown in Figure 5A, D and G. In the absence of nicardipine, only a small percentage of cells at any depth displayed measurable accumulation of the fluorescent paclitaxel derivative. Pretreatment of the TS^Adr^ with 10 *μ*M nicardipine inhibited P-gp and produced a striking increase in the intracellular accumulation of BODIPY-taxol. The distribution pattern was identical to that observed in the non-P-gp-expressing TS^WT^. At a distance of 40 *μ*M below the periphery, the fluorescence was relatively evenly spread along the ‘*xy*-plane’ in nicardipine-treated TS^Adr^ (Figure 5D, F). However, at the greatest depth examined, there was a marked drop in the distribution of BODIPY-taxol (Figure 5G, I) in the central region along the *xy*-plane. This suggests an incomplete penetration of the compound; however, increasing the exposure time to BODIPY-taxol from 2 to 6 h produced a relatively homogeneous distribution at this depth (data not shown), similar to that described for the TS^WT^ in Figure 3H. The accumulation of calcein-AM (Figure 6A–C

) and rhodamine 123 (Figure 6D–F) was also quantified in TS^Adr^ in the presence (solid lines) or absence (dashed lines) of nicardipine pretreatment. The extent of accumulation for both the compounds at each depth examined was significantly increased in TS^Adr^ by the nicardipine pretreatment. There were some differences in the distribution patterns observed for calcein-AM and rhodamine 123; however, they were only apparent at greater depth within the tissue. For example, at −40 *μ*M the fluorescence intensity of calcein-AM was considerably lower in the region 200–300 *μ*M along the *xy*-plane (Figure 6B), compared to that observed for rhodamine 123 (Figure 6E). The difference was less pronounced at −80 *μ*M (Figure 6A, D). However, fluorescence was clearly detectable for rhodamine 123, but not for calcein-AM, in this central region at this particular depth within the TS. The distribution patterns of rhodamine 123 and BODIPY-taxol were similar in TS^Adr^ treated with 10 *μ*M nicardipine, and indicate an extensive penetration through the tissue. In contrast, the calcein-AM distribution was confined to the outer few cell layers and this was identical to that observed in TS^WT^. The effect of P-gp inhibition did, however, increase the extent of accumulation in this surface localised cell population.

## DISCUSSION

Efficient chemotherapy in solid tumours relies on achieving an appropriate pharmacokinetic lifetime, which in turn is dependent on two early conditions being met: (i) extensive distribution throughout avascular regions and (ii) sufficient accumulation within the discrete cell populations. Both factors may be altered in cancerous tissue due to the presence of high interstitial pressure, extensive extracellular matrix, cell–cell contact and the expression of efflux pumps such as P-gp on the cell surface. However, unlike the case in haematological disorders, the role of P-gp in altering drug pharmacokinetics in solid tumours has been questioned (Kaye, 1995,^1998^).

A major basis for the ambivalence towards a role for P-gp has arisen due to the significant barriers to distribution produced by the 3-D organisation of cells in solid tumours, even in the absence of this drug efflux pump. For example, doxorubicin displays a highly localised distribution to the outer cell layers of solid tissue, yet the accumulation in this region is extensive (Durand and Olive, 1981; Durand, 1986; Wartenberg and Acker, 1996). In contrast, paclitaxel exhibits a wider distribution in tumour models; however, the rate of its permeation through the tissue is slow (Nicholson *et al*, 1997; Kuh *et al*, 1999). Cisplatin is poorly accumulated within cells, yet its penetration through TS models is relatively extensive (Erlichman *et al*, 1985). To date, investigations on the role of P-gp in modulating drug distribution in solid tumours have focussed on doxorubicin, mainly due to its availability in radiolabelled form or its inherent fluorescence. In the present paper, a selection of compounds with different chemical characteristics were chosen to examine distribution in the TS model, which provides a reflection of the avascular nodules found in solid tumours *in vivo* (Thomlinson and Gray, 1955; Harris, 2002). The use of confocal microscopy permitted investigation in a noninvasive fashion compared to previously used techniques such as autoradiography (Nederman *et al*, 1988; Kobayashi *et al*, 1993) or flow cytometry (Durand, 1990). None of the compounds used was an anticancer agent; however, the fluorescent compounds rhodamine 123 and calcein-AM are both well-established substrates for transport by P-gp (Homolya *et al*, 1993; Shapiro and Ling, 1998). The data using purified reconstituted P-gp revealed that BODIPY-taxol stimulated ATP hydrolysis and displaced the binding of [^3^H]azidopine, both indicative of a direct effect on the protein. Moreover, this highly fluorescent derivative of paclitaxel retained the ability to cross the plasma membrane of MCF7^WT^ cells and thus served as a marker to assess the influence of P-gp in TS. The confocal images of each compound were confined to a depth of 80 *μ*m from the surface, since the fluorescent signal is subject to attenuation due to absorption and scattering artefacts at greater depths (Wartenberg *et al*, 1998). This depth of field does extend sufficiently into the tissue to provide measurements of distribution in both the outer proliferative and deeper quiescent cell populations (Hall *et al*, 2003; Walker *et al*, 2003).

Durand (1990) proposed that the effect of P-gp on drug distribution would manifest as an increased penetration rate through tissue. This effect would result from a lack of significant reduction in extracellular concentration during passage due to the reduced accumulation in cells. This hypothesis was supported by the reduced penetration of [^14^C]doxorubicin through TS following inhibition of P-gp, thereby producing toxicity in perivascular cells while preventing access to central quiescent cells (Tunggal *et al*, 2000). However, doxorubicin distribution may not provide a ‘global’ or typical template for drug distribution due to the sequestration within the outer cell population caused by its avid binding to cellular macromolecules (Sutherland *et al*, 1979; Kwok and Twentyman, 1985; Tunggal *et al*, 1999). The distribution of fluorescence produced following calcein-AM addition displayed a similar pattern to doxorubicin, with localisation restricted to the outer few cell layers. The lack of distribution at deeper regions of TS is unlikely due to physical artefact caused by nonspecific quenching of fluorescence since it was not evident for either rhodamine 123 or BODIPY-taxol. Inhibition of P-gp in TS^Adr^ by nicardipine produced a large increase in calcein-AM accumulation; however, the overall distribution remained restricted to similar regions as in TS^WT^. P-gp is expressed at deeper layers of TS^WT^ Walker *et al*, 2003) although the levels are very low, and it was thought that this may account for the lack of calcein fluorescence in this region. However, nicardipine addition to TS^WT^ did not affect calcein-AM distribution at any depth of the TS^WT^. The increased accumulation of BODIPY-taxol or rhodamine 123 in deep regions of TS^Adr^ in the presence of nicardipine suggests that the P-gp inhibitor displays significant activity and penetration in the central regions of the tissue. The highly localised distribution pattern may be due to the large diffusion gradient for calcein-AM into cells in the TS periphery being constantly maintained by the rapid cleavage to calcein, thereby appearing to ‘sequester’ fluorophore within this cell layer (Homolya *et al*, 1993).

The results presented for calcein-AM and those for doxorubicin (Wartenberg and Acker, 1996; Tunggal *et al*, 2000) show that P-gp is able to prevent the accumulation of compounds with a relatively ‘restricted’ distribution pattern. Does it afford a similar effect on the penetration of compounds with different physico-chemcal properties? Unlike doxorubicin, the penetration of another anticancer agent, paclitaxel, is extensive throughout 3-D histocultures of patient tumours (Kuh *et al*, 1999) and the multicellular layer model (Nicholson *et al*, 1997). However, the rate of penetration through the tissue is thought to be considerably slower than doxorubicin or tirapazamine (Nicholson *et al*, 1997; Phillips *et al*, 1998). These investigations were facilitated by the availability of radiolabelled paclitaxel; however, the measurement of [^3^H]drug distribution by autoradiography is limited by the need to fix the tissue, which may itself affect the drug distribution profile. To counter this problem, a confocal microscopy approach was used to measure the distribution of BODIPY-taxol in TS. BODIPY-taxol has previously been demonstrated to interact with microtubules and this association is competitively inhibited by the parent compound paclitaxel (Bicamumpaka and Page, 1998; Melan, 1998). This compound may therefore be considered to provide a faithful representation of paclitaxel actions in a cellular environment. This was borne out by the finding that BODIPY-taxol labelled numerous cytotosolic sites including the nuclear membrane in MCF7^WT^ cells both in monolayer and 3-D cultures, similar to the intracellular localisations previously reported (Bicamumpaka and Page, 1998). As described above, the fluorescent paclitaxel derivative behaves as a transported substrate of P-gp, and consequently did not accumulate significantly at any location in TS^Adr^, a situation analogous to that observed in monolayer cultures (Martin *et al*, 1999). Further proof of a direct involvement for P-gp in maintaining lower cellular drug concentrations was the restoration of accumulation, by inhibition of P-gp, to levels seen in TS^WT^. In addition, the distribution of BODIPY-taxol in nicardipine pretreated TS^Adr^ was relatively homogeneous at all tissue depths examined, indicating that effective inhibition of P-gp is possible within a solid tissue mass. The pivotal role for P-gp in determining drug pharmacokinetic profiles has also been demonstrated in normal noncancerous tissue through prevention of paclitaxel and BODIPY-taxol passage across the blood–brain barrier (Fellner *et al*, 2002).

In summary, the results presented in this paper show that the expression of P-gp in 3-D organisation of cells does affect the overall accumulation of a variety of compounds. While the penetration or distribution of compounds throughout solid tissue is strongly dependent on the physico-chemical properties of a drug (e.g. doxorubicin *vs* paclitaxel), a reduction in accumulation within specific cellular compartments by efflux pumps such as P-gp will also contribute to the global pharmacokinetic characteristics. Thus, it seems that the notion of dismissing P-gp to ‘bystander’ status in drug-resistant solid tumours appears unfounded, and some attention to overcoming its actions in chemotherapeutic strategies will remain a priority.
